# Preparation of Spheroids from Primary Pig Cells in a Mid-Scale Bioreactor Retaining Their Myogenic Potential

**DOI:** 10.3390/cells11091453

**Published:** 2022-04-25

**Authors:** Katja Stange, Amir Keric, Andreas Friese, Monika Röntgen

**Affiliations:** 1 Institute of Muscle Biology and Growth, Research Institute for Farm Animal Biology (FBN), 18196 Dummerstorf, Germany; stange.katja@fbn-dummerstorf.de; 2 OMNI Life Science GmbH & Co. KG, 28359 Bremen, Germany; amir.keric@ols-bio.de (A.K.); andreas.friese@ols-bio.de (A.F.)

**Keywords:** spheroid, myosphere, C2C12, porcine muscle, mid-scale bioreactor, 3D cell culture

## Abstract

Three-dimensional cell culture techniques mimic the in vivo cell environment more adequately than flat surfaces. Spheroids are multicellular aggregates and we aimed to produce scaffold-free spheroids of myogenic origin, called myospheres, using a mid-scale incubator and bioreactor hybrid. For the first time, we obtained spheroids from primary porcine muscle cells (PMCs) with this technology and compared their morphology and growth parameters, marker expression, and myogenic potential to C2C12-derived spheroids. Both cell types were able to form round-shaped spheroids in the bioreactor already after 24 h. The mean diameter of the C2C12 spheroids (44.6 µm) was larger than that of the PMCs (32.7 µm), and the maximum diameter exceeded 1 mm. C2C12 cells formed less aggregates than PMCs with a higher packing density (cell nuclei/mm^2^). After dissociation from the spheroids, C2C12 cells and PMCs started to proliferate again and were able to differentiate into the myogenic lineage, as shown by myotube formation and the expression of F-Actin, Desmin, MyoG, and Myosin. For C2C12, multinucleated syncytia and Myosin expression were observed in spheroids, pointing to accelerated myogenic differentiation. In conclusion, the mid-scale incubator and bioreactor system is suitable for spheroid formation and cultivation from primary muscle cells while preserving their myogenic potential.

## 1. Introduction

Since its first implementation over 100 years ago [1], the 2-dimensional animal cell culture has made enormous advances and enabled a plethora of remarkable achievements in all fields of biomedical sciences. It allowed for life-saving technologies such as drug development, vaccination, and regenerative medicine [2]. Now we begin to understand that 3-dimensional approaches are more powerful and indispensable for a wide range of topics. It has been known for a long time that flat surfaces do not adequately mimic the physiological environment of cells, mainly the extremely complex extracellular matrix (ECM), mechanical and biochemical signals, and intercellular communication [3,4]. Thus, monolayer cultures often fail to produce results transferable to in vivo experiments. Especially in the view of attempting to reduce animal experiments, suitable 3D culture techniques with higher physiological relevance become even more important, e.g., for studies on (stem) cell differentiation, drug screening, cellular cytoskeleton, adhesion, or signaling [5,6].

A variety of experimental approaches have been implemented, such as explant, microcarrier, spheroid, or organoid culture [4,7]. Many studies have attempted to produce and culture spheroids—multicellular aggregates usually characterized by high cell density and a spherical shape [4,8,9]. Since spheroids recapitulate the natural environment of cells in a superior way, they might be suitable for various crucial applications in basic research and in applied sciences, such as tissue engineering or transplantation (reviewed in [9,10]). Using spheroids, we can answer questions regarding the self-organization and self-assembly of cells. Spheroids mimic the tumor architecture better and allow for studying drug efficacy and transport [11,12] and might even be used as building blocks for organ printing in the future [9]. Besides cancer spheroids and neurospheres [11,13], a variety of other cell types, such as endothelial and mesothelial cells, cardiomyocytes, hepatocytes, and chondrocytes, have been studied after spheroid formation [9,14,15,16].

Compared to other 3D culture systems, spheroid culturing is relatively simple, scalable (high throughput), reproducible, and allows for co-culture of cells [7], although many challenges remain, e.g., homogeneity, viability, or influencing protein expression [8]. Most spheroids are grown in suspension culture, e.g., on non-adhesion materials such as agarose, in hanging drops, or rotating vessels [4,8,9,17]. Thus, gravity forces, preventing cell adhesion and enhancing cell–cell contacts, are used to promote aggregation [9]. All of these methods are constantly improved and can be used for cell lines as well as for primary cells, but have to be carefully adapted [9]. 

Bioreactors can be used in many ways and offer the advantage of easy standardization and reproducibility [18], e.g., when compared to hanging drop techniques. In addition, individual cellular demands can be satisfied by adjusting the culture conditions (e.g., media, oxygen supply, and mechanical force). Moreover, bioreactors are less labor-intensive than other techniques, such as the hanging drop method.

Different organic and inorganic matrices, scaffolds, sponges, (hydro)gels, and microcarriers are used to support 3-dimensional growth of cells by providing them with a micro architecture [5,19]. As with a cell culture medium, the optimal composition of such materials can be very individual and complex, and it should be considered that additional (unphysiological) components are introduced into the cell culture. Biomaterials potentially promote cell activity and function. In the case of artificial biomaterials their characteristics can be easily controlled during the production process [20], but the cell-type-specific design is impeded when cellular needs, e.g., in the stem cell niche, are not fully understood [21]. Natural biomaterials on the other hand have a high biocompatibility, but unfortunately can be immunogenic, hardly available depending on the source, and contain contaminants [20,22]. Thus, scaffold-free spheroid formation can be advantageous, if it can be established for the desired cell-type and application.

Spheroid formation is triggered by direct contact of cells with long-chain ECM fibers, leading to lose aggregation. Subsequently, cadherin expression is upregulated, and the proteins accumulate at the surface. Cadherin proteins will then tightly bind to each other, as the aggregates become more densely packed [23]. During spheroid growth, molecular gradients develop due to limitations in diffusion. Thus, nutrients, metabolites, gases, and growth factors are not evenly distributed and areas of waste buildup can be present [8,9,10]. Therefore, the most proliferative cells are found at the surface, whereas quiescent and dead cells are more likely to be found in the spheroid core [12].

Skeletal muscle is the most abundant tissue in the body [24]; still, a myriad of research questions remain unanswered and numerous pathogenic conditions are waiting for causal therapy. Therefore, spheroids of myogenic origin, called myospheres, are an interesting research topic. Muscle has a very complex structural organization and function, which cannot be fully recreated in 2D culture [25]. In addition, it is very challenging to maintain myogenic stem cells, called satellite cells, in a conventional cell culture after isolation. When being removed from their stem cell niche, they easily lose their stemness and undergo premature differentiation into multinucleated myotubes [25]. It was reported that cells derived from myospheres show a myoblast-like behavior and are able to form both new satellite cells and new myofibers [25,26,27,28]. Their regenerative capacity might be even higher compared to single myogenic cells [25,29]. Several studies showed that in 3D culture, cells have a prolonged cultivation period, myotube size, and the protein content is enhanced, and myogenic maturation seems to be improved (reviewed in [25]). This underlines the enormous potential of myospheres in regenerative medicine and tissue engineering.

Table 1 shows an exemplary selection of previously described methods for the generation of spheroids from cells of myogenic origin or with myogenic properties. 3D cultivation using myogenic cells, but not building spherically shaped myospheres using, e.g., 3D scaffolds for a myobundle system or myotube alignment, were included (for a review, see [25]). The formation and cultivation of myogenic spheroids in a static suspension culture using culture flasks or plates has been reported frequently. Free-floating cultures were described with murine and human muscle cell lines [30,31,32] as well as with primary muscle cells from mice [27,29,33], humans [34,35,36], and dogs [37]. The majority of primary muscle-derived cells (MDC) will adhere quickly, allowing the use of the pre-plating technique for enrichment [33]. Suspension culture benefits from the observation that a small subpopulation of cells is slow- or even non-adherent and, thus, able to form free-floating clusters under low-attachment conditions [33,34]. These initial myospheres can be passaged by trapping and/or by mechanical or enzymatic dissociation [25]. The incorporated cells retain their ability to proliferate and (trans)differentiate, are able to fuse with other cells, adhere under appropriate conditions, and show regenerative potential in vivo [29,33]. The 3D cultivation of myogenic cells in a dynamic suspension culture, e.g., using spinner flasks or bioreactors, has been managed as well, although the biomaterials have been mostly used as scaffolds [31,38,39].

In our study, we focused on the scaffold-free formation and expansion of myospheres using a mid-scaled, paralleled, automated bioreactor with bi-directionally rotating tubes. The CERO 3D Incubator and Bioreactor (OLS, Bremen, Germany), formally known as Bio-Levitator (Global Cell Solutions, Charlottesville, VA, USA, and Hamilton Company, Reno, NV, USA [19]), was used here. It includes an incubation chamber with four independently fillable and controllable tubes. Due to small homogenization fins, no impeller needs to be inserted into the tubes, which substantially reduces the risk of contamination. The bi-directional rotation and the resulting levitation within the tubes keeps the 3D aggregates floating, thereby minimizing shear forces and cell stress, making it suitable for sensitive (stem) cell populations. This bioreactor system has been successfully used for spheroid formation from human (induced) pluripotent stem cells [42,43], hepatic HepG2 and Huh7 cells, and murine embryonic stem cell-derived cardiomyocytes [44].

For the first time, we successfully produced and cultivated spheroids from primary porcine muscle cells (PMCs) with this technology and compared their features to spheroids derived from myoblasts of the C2C12 cell line. Furthermore, we show the myogenic potential of cells derived from spheroids.

## 2. Materials and Methods

### 2.1. Cell Culture

Four-day-old German Landrace pigs raised in the experimental pig unit of the Research Institute of Farm Animal Biology (FBN) were slaughtered following the guidelines of the Animal Care Committee of the State Mecklenburg-Western Pomerania, Germany, based on the German Law of Animal Protection. As animals were not manipulated before slaughter, no animal experiment was conducted. Porcine muscle cells (PMCs) were isolated from semimembranosus and longissimus dorsi muscles as described previously [45]. In brief, dissected tissues were trimmed, enzymatically digested with trypsin (4000 U/mL, Sigma-Aldrich, St. Louis, MO, USA), and purified via 40/70% Percoll gradient centrifugation. Cells were seeded on collagen type I coated dishes (Greiner bio-one, Frickenhausen, Germany) and cultivated for 3–4 days in growth medium (αMEM Eagle containing 2.5 µg/mL amphotericin B, 0.05 mg/mL gentamycin, 100 U/mL penicillin/streptomycin (all from PAN Biotech, Aidenbach, Germany), and 20% fetal bovine serum (FBS, Gibco). Cells were harvested using 0.05% trypsin/0.02% EDTA (PAN Biotech) and cell number and viability were determined (Countess Automated Cell Counter, Thermo Fisher, Darmstadt, Germany). Subsequently, cells were frozen and stored in liquid nitrogen until use.

C2C12 cells were obtained from ATCC [46] and cultivated in growth medium (DMEM 4.5 g/L glucose, 10 U/mL penicillin/streptomycin (all from PAN Biotech), and 10% FBS (Gibco). Cells were passaged 3 times a week before reaching 80% confluency using trypsin/EDTA.

Cultivation of all cells was conducted under a humidified atmosphere with 5% CO_2_ at 37 °C.

### 2.2. Suspension Culture for Spheroid Formation

PMCs were thawed and cultivated for 3–4 days before use. C2C12 cells as well as PMCs were consecutively washed with DPBS without Ca^+^/Mg^2+^ (PAN Biotech), 0.25 mM EDTA in DPBS, and 0.5 mM EDTA in DPBS and harvested using an animal-component-free enzymatic dissociation solution (Stemcell Technologies, Cologne, Germany). Cells were strained over a 50 µm CellTrics filter (Sysmex, Norderstedt, Germany) to avoid agglutination.

For subsequent suspension culture and spheroid formation, the CERO 3D Incubator and Bioreactor (OLS) was used. Harvested cells were seeded into CEROtubes (C2C12: 200,000 cells/mL, PMCs: 100,000 cells/mL, 10 mL per tube). Cell numbers were selected due to our own personal experience with the respective cell types and from results of preliminary experiments. The program used during the initial inoculation phase and the following culture phase can be seen in Table 2. No agitation period was used. Cells/spheroids were cultured for a maximum of 7 days.

### 2.3. Quantitative Analyses of Spheroids

Throughout cultivation in the CERO 3D Incubator and Bioreactor, representative samples were taken on given days of analysis to determine the growth parameters of spheroids. First, 500 µL of cell suspension were transferred into a 24-well plate and 10 random pictures were taken using a Primovert microscope (Zeiss, Jena, Germany). Spheroid diameter was determined using Fiji [47,48]. On average, 1295 spheroids (minimum of 601) were analysed per cell type and day of analysis, originating from three independent experiments. In total, 10 µL of suspension were transferred in another well and the number of aggregates in 4 drops per day of analysis was counted manually (spheroid number). After analysis, samples were returned to the culture. Three independent experiments per cell type were conducted. Samples of the spheroids at Days 1, 3, and 7 of culturing were also stained with Hoechst 33528 (Sigma-Aldrich), examined under a Leica DM4000B microscope, and the number of cell nuclei per spheroid area was determined (packing density). On average, 29 spheroids (minimum of 26) were analysed per cell type and day of analysis, originating from two independent experiments.

### 2.4. Histological Analysis of Spheroids

At Day 7 of cultivation, the spheroids were removed from the CEROtube and snap frozen in liquid nitrogen using an OCT Embedding Matrix (Carl Roth, Karlsruhe, Germany) and Tissue-Tek cryomolds (Sakura Finetek, Staufen im Breisgau, Germany). Spheroids can be stained with 0.1% Eosin for better visibility before freezing. Transverse, serial 10 μm sections were cut with a cryotome (Leica Biosystems, Brünn, Germany) at −20 °C. Slides were stained with Haematoxylin and Eosin (HE) and imaged with an Olympus BX43 microscope.

For visualization of collagen type 1 and 3, cryosections were fixed with paraformaldehyde, permeabilized with 0.1% TritonX100 in PBS for 20 min, and blocked in PBS containing 20% goat serum (Biowest, Nuaillé, France) and 0.1% TritonX100 for 1 h. Sections were then incubated overnight with a rabbit anti-collagen 1/3 antibody (Thermo Fisher Scientific, Darmstadt, Germany) diluted 1:25 in 6% goat serum in PBS. A goat anti-rabbit IgG 488 (1:1000, Alexa Fluor, Life Technologies, Darmstadt, Germany) was used, and after washing again with DPBS, cell nuclei were stained with DAPI (1 µg/mL, Sigma-Aldrich). Microscopy was performed with a Leica DM4000B and the software cellSens (Ver. 3.2, Olympus, Hamburg, Germany).

In addition, cryosections were stained for Desmin, F-Actin, and Myosin heavy chain using antibody-assisted immunofluorescence staining as described previously [49].

### 2.5. Analysis of Spheroid Viability

Protocols to analyse spheroid viability were performed as described in [50]. To test for the presence of viable, apoptotic, and dead cells, spheroids were natively stained after 7 days of cultivation. Spheroids were removed from the CEROtubes, centrifuged (5 min, 30× *g*), and carefully resuspended in growth medium. For the control, spheroids were incubated in 70% methanol for 30 min. For the live dead assay, spheroids were stained with 2 µM Calcein-AM (Biolegend), 5 µM propidium iodide (PI, Invitrogen/Thermo Fisher Scientific, Darmstadt, Germany), and 20 µg/mL Hoechst 33258 in DPBS without Ca^+^/Mg^2+^ for 20 min (instead of 30 min as in [50]) at 37 °C, washed with growth medium, and incubated for another 10 min at 37 °C in growth medium. For the apoptosis assay, spheroids were washed with Annexin binding buffer (10 mM HEPES, 140 mM NaCl, 2.5 mM CaCl_2_ in H_2_O), stained with Annexin V Alexa Fluor 488 (1:20, Thermo Fisher Scientific), 5 µM PI, and 20 µg/mL Hoechst 33258 in Annexin binding buffer for 30 min at 37 °C, and washed again with Annexin binding buffer. Confocal microscopy was performed immediately at LSM800 (Zeiss, Jena, Germany) with the corresponding ZEN software.

### 2.6. Myogenic Differentiation of Cells from Spheroids

Spheroids were removed from the CEROtubes after 5 days of cultivation. To first test for the outgrowth of viable cells, spheroids were transferred to a Matrigel-coated (growth factor reduced, BD Biosciences) 4-well plate (Nunclon Delta surface, Thermo Fisher Scientific) and allowed to adhere in growth medium. For the myogenic cell differentiation assay, spheroids were washed with 0.2 mM EDTA in DPBS and dissociated with trypsin/EDTA for 15 min at 37 °C to obtain a single cell suspension. The procedure to obtain single cells from the spheroids has been described by Fischer et al. [43] and was used with small modifications.

Then, cells were seeded on Matrigel-coated 4-well plates and cultivated in growth medium. Before reaching confluence, differentiation was induced by reducing the FBS content in the medium to 2%. After 6–7 days, myotube formation was observed and cells were fixed with 4% paraformaldehyde for subsequent immunofluorescence staining. Cells were permeabilized in 0.5% Triton X100 for 20 min and blocked in 20% goat or rabbit serum in 0.5% Triton X100 for 1 h. The following primary antibodies were diluted in DPBS containing 0.5% Triton X100 for incubation: rabbit anti-Ki67 (1:100, IgG, NovusBio, Wiesbaden, Germany), mouse anti-Desmin (1:80, IgG1, DAKO, Glostrup, Denmark), mouse anti-MyoG (1:50, IgG1, abcam, Cambridge, Great Britain), and mouse MF20 (undiluted, IgG2b, DSHB). After washing with DPBS, the following secondary antibodies were used (all 1:1000, Alexa Fluor, Life Technologies/Thermo Fisher Scientific): goat anti-mouse 488, goat anti-rabbit IgG 488, goat anti-mouse IgG2b 488, goat anti-mouse IgG1 546, and rabbit anti-mouse IgG 488. F-Actin was visualized via incubation with Phalloidin Cruz Fluor 594 conjugate (1:1000, Santa Cruz Biotechnology, Santa Cruz, CA, USA) for 1 h. After washing again with DPBS, cell nuclei were stained with DAPI (one µg/mL, Sigma-Aldrich). Microscopy was performed with a Leica DM4000B and the corresponding software Leica QWin V3.

### 2.7. Statistical Analyses

Spheroid diameter, number, and packing density are shown as Box-and-Whisker plots, showing the median and the maximum 1.5 of the interquartile range (Q1–Q3); outliers are presented as circles. For statistical analyses, SigmaPlot 13.0 (Systat Software Inc., San Jose, CA, USA) was used as follows: (1) spheroid number at Days 1, 2, 5, and 6: one-tailed Student’s t-test (normality (Shapiro–Wilk) and equal variance (Brown–Forsythe) test passed); (2) median of spheroid diameter, packing density, and spheroid number at Days 3, 4, and 7: Mann–Whitney Rank Sum Test (normality (Shapiro–Wilk) or equal variance (Brown–Forsythe) test failed); and (3) spheroid diameter: Kruskal–Wallis One Way ANOVA on Ranks (All Pairwise Multiple Comparison Procedures (Dunn’s Method); normality (Shapiro–Wilk) test failed). Statistical significances were assigned as follows: *** *p* ≤ 0.001, ** *p* ≤ 0.01, * *p* ≤ 0.05, trend *p* ≤ 0.1. The frequency distribution of spheroid diameter was calculated according to quartiles with IBM SPSS Statistics 22. In total, 6964 (C2C12) or 11,160 (PMCs) spheroids were included in the calculation. For quantitative analyses, representative images are shown.

## 3. Results

### 3.1. Formation of Spheroids from Muscle Cells in the CERO Incubator and Bioreactor

Primary myogenic precursor cells derived from porcine muscle and the conventional murine myoblast cell line C2C12 were compared regarding their ability to form 3-dimensional spheroids in a suspension culture. 

**Figure 1:**
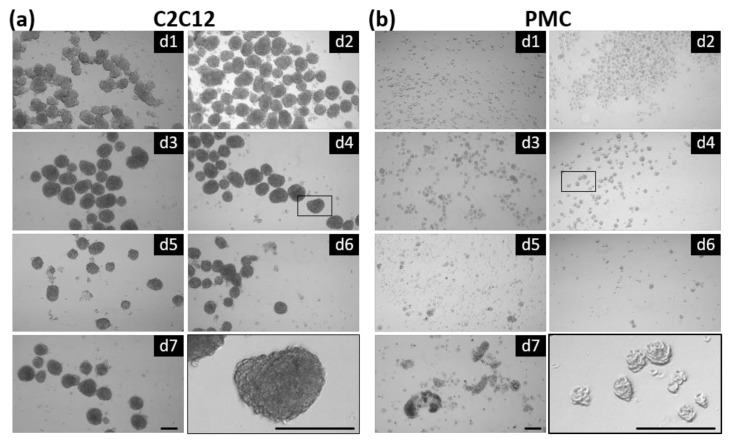
Myosphere formation in suspension culture. C2C12 (**a**) and primary pig muscle cells (PMCs, (**b**)) were seeded (200,000 or 100,000 cells/mL, respectively) into the CERO 3D Incubator and Bioreactor (OLS) and spheroid formation was analyzed for 7 days. A considerable number of round-shaped spheroids was seen at all time points. The image on the bottom (right side) shows a magnification of spheroid(s) at Day 4. Scale bar: 200 µm.

CERO 3D, a mid-scale incubator and bioreactor hybrid, was used for cultivation. The device uses up to four independent 50 mL CEROtubes with small fins on the wall to ensure gentle homogenization of the cell suspension and optimal gas exchange and nutrient supply within the 3D aggregates. After every rotation period, the rotation direction is changed (bidirectional rotation). During initial inoculation, the formation of aggregates takes place; in the following culture phase, the spheroids should expand. Both tested cell types were able to form spheroids in the CERO 3D device already after 24 h of incubation and remained stable for at least 1 week (Figure 1). The three-dimensional, spherically shaped structures show a homogenous morphology, although differences in size are visible. Some spheroids tended to adhere and fuse to each other, as seen, for example, in C2C12 cells at Days 3 and 6.

**Figure 2:**
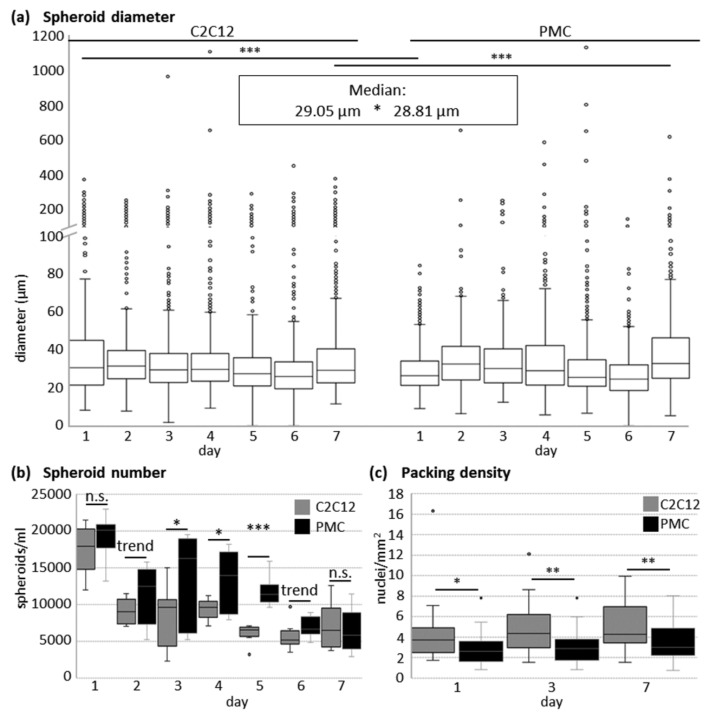
Quantitative analysis of the myosphere growth parameters. C2C12 and PMCs were seeded into CEROtubes and spheroid formation was analyzed for 7 days. (**a**) At Days 1 and 7, the diameter of the spheroids differs significantly between both cell types. The mean spheroid diameter over all days was significantly larger in C2C12 cells. (**b**) The number of spheroids formed by the C2C12 cells was significantly larger at Days 3, 4, and 5 and tended to be higher at Days 2 ad 6 as well. (**c**) Packing density of spheroids was calculated as the number of cell nuclei per spheroid area and is significantly higher in C2C12 cells at all the tested time points. Significant differences are marked by asterisks (*** *p* ≤ 0.001, ** *p* ≤ 0.01, * *p* ≤ 0.05). *p* ≤ 0.1 is a trend.

In the following, the quantitative parameters of the formed spheroids were analysed in more detail (Figure 2). The diameter of the single spheroids was quite variable (Figure 2a), with maximum values up to 1111 µm for C2C12 and 1135 µm for PMCs. Nevertheless, according to the frequency distribution, 50% of all spheroids had a size between 22 and 38 µm (C2C12) or between 22 and 39 µm (PMCs), respectively. The mean diameter of the C2C12 spheroids was significantly larger than that of the PMCs. The number of spheroids (Figure 2b) decreased during cultivation and the C2C12 cell cultures showed significantly less aggregates compared to the PMC cultures at Days 3–5 of culture. On Days 1, 3, and 7 after inoculation, the packing density of the spheroids was significantly larger in C2C12 cells (Figure 2c). In average, 2.89 nuclei/mm^2^ (PMCs) and 4.05 nuclei/mm^2^ (C2C12) were found, respectively (median). Packing density did not depend on spheroid size in our analysis (R^2^ = 7.68%).

### 3.2. Protein Expression of the Myogenic Markers and Extracellular Matrix in Spheroids

**Figure 3:**
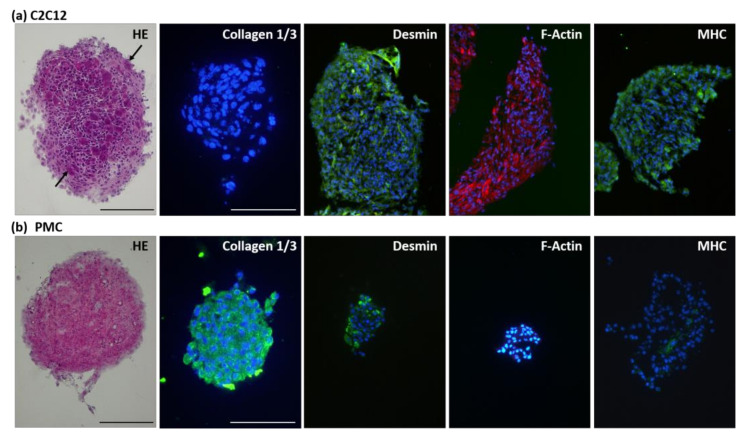
Histological analysis of the myospheres. After 7 days of cultivation, spheroids of C2C12 (**a**) and PMCs (**b**) were frozen and sectioned. Staining with Hematoxylin and Eosin (HE) allows for an overview regarding myosphere structure, arrows point to syncytia in C2C12 spheroids. The ECM proteins collagen 1/3 as well as the myogenic proteins Desmin, F-Actin, and Myosin heavy chain (MHC) were analysed via immunofluorescence staining; cell nuclei were stained with DAPI. Collagen 1/3 expression was detected in porcine spheroids only. All three myogenic markers were clearly detected in the C2C12 spheroids. In PMC spheroids only Desmin was widely expressed, whereas MHC expression was low and F-Actin seemed to be absent. Scale bar: 200 µm.

Cryosectioning of the spheroids followed by histological staining revealed further details on structure and protein expression (Figure 3). Hematoxylin–Eosin staining provides a useful overview of spheroid morphology and ECM structure. Cell nuclei are evenly distributed throughout the aggregates. Alignment of cells can also reveal the preceded fusion of two or more aggregates, which we often observed for C2C12 spheroids. Multinucleated syncytia were frequently observed in C2C12-derived spheroids (arrow). An antibody detecting both collagen type 1 and 3, showed expression of the proteins in the extracellular matrix (ECM) of PMC-derived spheroids. In C2C12 spheroids, the filamentous proteins Desmin, F-Actin, and Myosin heavy chain (MHC) were clearly expressed. Contrastingly, in PMC-derived spheroids only Desmin was widely expressed. Isolated MHC staining was observed, but no F-Actin protein.

### 3.3. Viability and Myogenic Potential of Spheroids

After successfully producing spheroids and assessing their morphology, we focused on the viability and functionality of the spheroids.

**Figure 4:**
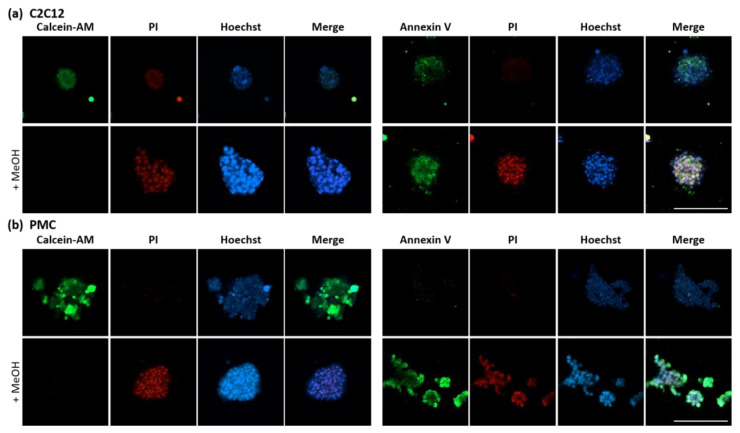
Cell viability in the myospheres. After 7 days of cultivation, spheroids of C2C12 (**a**) and PMCs (**b**) were directly stained and observed by confocal microscopy. Live or apoptotic cells were stained by Calcein-AM and Annexin V, respectively; dead cells were stained by PI. In control spheroids, apoptosis and cell death were induced by treatment with methanol. All spheroids were viable without signs of cell death. The C2C12 spheroids showed minor indications of apoptosis, which were not seen in PMCs. Scale bar: 200 µm.

To investigate viability, three common fluorescent dyes were used (Figure 4). The fluorescence of the Calcein-AM dye is activated by cellular esterases, which are active only in living cells. Annexin V will bind to phophatidylserines on the surface of intact but apoptotic cells, as well as inside dead cells. Propidium iodide (PI) intercalates with DNA and also binds to RNA, but cannot pass the intact membrane of viable cells, and is therefore widely used to stain dead cell nuclei. Methanol-treated controls show the absence of living cells and the appearance of apoptotic and dead cells as intended. C2C12 as well as PMC spheroids are viable, and no dead cells could be detected. A weak, diffuse background stain was observed in the C2C12 spheroids in the live dead assay. Nevertheless, the signal is clearly distinguishable from the PI-positive nuclei staining. In PMC spheroids, no Annexin V signal was detected, whereas in C2C12 spheroids weak apoptosis was seen, although substantially weaker than in the positive control.

**Figure 5:**
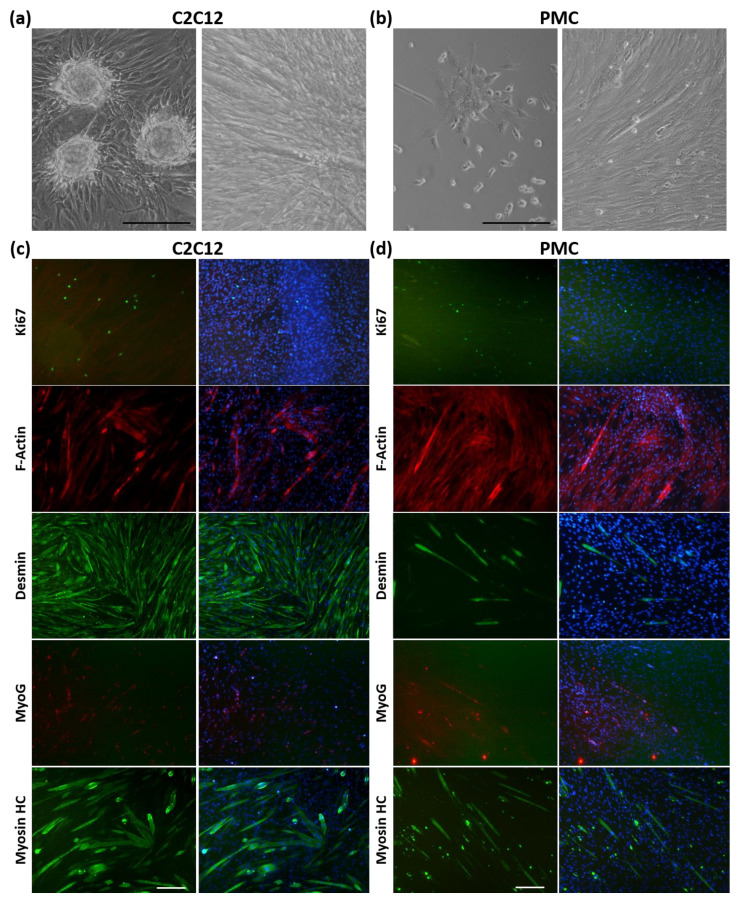
Myogenic differentiation of cells outgrown from myospheres. After 5 days of cultivation, spheroids were seeded, allowed to adhere, and C2C12 cells (**a**) as well as PMCs (**b**) were able to outgrow and form myotubes. For the differentiation assay (**c**,**d**), a single cell suspension was obtained from spheroids and cells were allowed to undergo myogenic differentiation in 2D culture. Subsequent immunofluorescence staining revealed proliferating (Ki67) and differentiating (MyoG) cells, as well as expression of the filament proteins F-Actin or Desmin. Multinuclear myotubes were formed by C2C12 (**c**) and PMCs (**d**) expressing Myosin heavy chain (HC). Right panel shows merged images with DAPI staining. Scale bar: 200 µm.

To assess the functionality of the spheroid cells, they were seeded into conventional 2D cell culture plates (Figure 5). The spheroids could easily attach to the surface and the cells grew numerously and quickly (Figure 5a,b). Cells from the dissociated spheroids were used for the differentiation assay (Figure 5c,d). The presence of Ki67+ cells indicates that proliferation of C2C12 cells and PMCs is taking place. A major physiological feature of muscle cells is their ability to undergo myogenic differentiation. Indeed, the myogenic differentiation potential was preserved during spheroid formation and cultivation as F-Actin, Desmin, and MHC staining show the formation of multinucleated myotubes. This process seems to be more intense in C2C12 cells. MyoG is expressed in the nuclei of both cell types as well.

## 4. Discussion

The future of cell cultures is 3-dimensional. Therefore, we aimed to evaluate the production and cultivation of myospheres from primary porcine muscle cells in comparison to the myogenic cell line C2C12. Spheroids have great potential for use in biomedical research, especially in investigating stem cells, since they mimic the cells’ physiological environment in a superior way compared to conventional 2D cultures. To the best of our knowledge, neither myospheres nor spheroids from other cell types of porcine origin have been produced to date. Our work opens the possibility of using a 3D model from myogenic pig cells in biological and medical/pharmacological studies. As the physiology of the pig resembles that of man in many respects [51,52,53,54], spheroids from pig cells have a major advantage over rodent models.

We used the CERO 3D Incubator and Bioreactor in our study. The cultivation program consisted of two phases with only slight differences between the PMCs and C2C12 cells. The same rotation speed of 80 rpm could be used for both cell types, which is a standard velocity also applicable for HepaRG cells and pluripotent stem cells [55]. An elongated inoculation phase with longer rotation period was necessary for the C2C12 spheroids, because aggregates tended to agglutinate and adhere to the surface. Therefore, the rotation pause in C2C12 during the culture phase was removed. These adjustments were not needed for the PMC spheroids, since neither increased agglutination nor adhesion was observed.

Culture medium composition affects cell growth and its characteristics, and thus can affect spheroid formation, composition, and properties [56]. However, the requirements of each cell type are very specific [57] and depend on their origin (species, tissue, cell line, or primary cell), metabolic needs, and their developmental and growth stage. Thus, to ensure the high cell quality needed as a prerequisite for preparation of high quantities of viable spheroids in a short time, the used media must be adapted to meet the cells’ needs. Therefore, we selected appropriate basal media generally used for expansion of C2C12 cells (DMEM high glucose) or primary PMCs (αMEM) for our study [46,58,59,60]. Like cancer cells [61], cells of the murine C2C12 line have a more glycolytic metabolism, therefore needing more glucose and amino acids for growth [62,63,64]. In contrast, low glucose levels as in αMEM promote proliferation and self-renewal but delay premature differentiation in PMC [61], thereby being beneficial for spheroid generation in our study. Much more experimental work will be needed to learn how targeted changes in media components can be used to modulate the spheroid properties for different purposes. FBS, being a complex mixture with mostly unknown composition [57], was used from the same batch for both cell types to ensure reproducibility.

Although both cell types tested here are strongly adhesive in 2D culture, round-shaped spheroids could be successfully produced in a very short time without the need to provide additional structures (e.g., a scaffold), clearly being one of the strengths of this study. Several scaffold-free and scaffold-based approaches as well as combinations thereof are used in 3D cell culture to recapitulate ECM properties [65]. For instance, collagen, gelatin, alginate, chitosan, or agarose are frequently used and their unique properties can be combined as needed [66]. Indeed, biomaterials can be helpful or necessary to enhance cell viability and to regulate cell activity and function [66]. However, all biomaterials have to be obtained in sufficient amount and quality, which can be quite laborious; for example, if recombinant protein production or acquisition from humans or other organisms is involved [66]. Moreover, (immunological) compatibility has to be ensured for use in transplantation medicine. The ECM defines the mechanical properties of a tissue [67]. It is of utmost importance for cell biological functions, such as cell survival, proliferation, migration, or fate, and is able to store growth factors to regulate extracellular signaling [14,67,68]. In muscle ECM, collagens, glycoproteins, proteoglycans, and elastin can be found [69]. Collagens are crucial to regulate stiffness and cohesiveness [68] and, because of their high abundance, are widely used as scaffold material in 3D tissue engineering of muscle, skin, bone, or in cancer research [66]. Collagen hydrogel has been shown to improve cell proliferation and differentiation in spheroids derived from human MSCs and HUVECs [66,70]. For some applications, such as bridging of non-healing bone defects or cartilage regeneration using chondrocytes [51], eliminating scaffolds completely will be hardly possible in the near future. However, scaffold-free technology is beneficial and can overcome disadvantages going along with natural or synthetic materials (immunogenicity, bioavailability, toxicity, and biodegradability) [66]. We examined collagen type 1 and 3, since these two collagen types make up approximately 75% of total muscle collagen, and are known to regulate strength and elasticity, respectively [69]. Remarkably, collagen type 1 and 3 expression was found in PMC-derived spheroids. This shows that these primary myogenic cells are able to produce ECM components, which are indispensable in vivo, in the mid-scale bioreactor system used in this study. We consider this a huge advancement, although further investigations on ECM components will have to follow.

Spheroid size depends on the selected culture system, culture condition and duration, and the cell type used. When grown in a non-adhesive static culture, loose aggregation of C2C12 cells was reported already 3–4 h after seeding, but compact spheroids occurred only at Day 4 of culture [30]. Primary murine cells also needed several days to form compact myospheres [29]. The CERO 3D technology seems to be suitable to accelerate the process of spheroid formation, since we observed compact structures already after 24 h. Furthermore, we were able to reach a comparable spheroid size. For C2C12, a size of 80–200 μm was reported for mature spheroids in a static culture [2], which was reached for numerous spheroids in our study within 1 week as well. Since the production and cultivation of porcine myospheres has not been described before, we refer to other primary myogenic cells and find comparable diameters. Free-floating myospheres derived from murine hindlimb muscle reached an initial size of 25–50 µm and grew up to 300–400 µm during static culture [29]. Canine myospheres reached a comparable size of 25–50 µm within the first 10 days and later on grew up to 200–400 µm [25,37]. According to our own experience with the respective cell type and preliminary experiments, we used different cell numbers for initial seeding into the bioreactor for C2C12 cells and PMCs, which has to be taken into account when comparing the quantitative features of the formed spheroids. Nevertheless, each cell type has its specific growth kinetic, even when the same cell number would have been used. For C2C12 cells or other cell lines, the availability of cells is mostly no problem, but when working with primary cells, the bioavailability is always a bottleneck. Thus, it is very beneficial that for PMC, a substantially lower cell density can be used while still achieving high-quality spheroids in a short time. Remarkably, C2C12 cells formed less spheroids than PMC, although a higher cell number was used. Appropriately, C2C12 spheroids were larger and more densely packed.

Although, we did not investigate the long-term cultivation of C2C12 and porcine myospheres in our cultivation system until now, it was shown to be feasible in static cultures using C2C12 cells (more than 20 days, [30]), murine primary cells (59 days, [29]), and canine primary cells (at least 2 weeks, [37]). Therefore, we assume that longer cultivation is possible in the bioreactor as well. We found no proliferating cells (Ki67+) in C2C12 and PMC spheroids after 7 days of culture (data not shown). Consequently, cell proliferation and growth of spheroids was not observed in both tested cell types and packing density was not substantially altered during cultivation. In accord to that, even in younger C2C12-derived spheroids (24 h) the absence of the proliferation marker Ki67 was reported [30].

Unless in 2D culture, cell movement can be limited and dead cells cannot detach into the medium from the inside of the spheroid [8]. Besides the absence of proliferating cells in C2C12 and PMCs, the spheroids were in good condition as no apoptotic or dead cells were seen, even 1 week after the seeding and start of spheroid formation. In a static cell culture, the transport of nutrients, gases, and waste products is achieved by passive diffusion only. This is not suitable for constructs larger than 200 µm [71]. Small molecules, e.g., oxygen, can pass a distance up to 150–200 µm via diffusion in MSCs [23]. The flow produced in a bioreactor improves the supply of 3-dimensional structures and thus positively influences cell viability [71]. For instance, in contrast to a static culture, no necrotic core was observed in human MSC grafts in a rotating bioreactor [72].

C2C12 cells become quiescent during spheroid culture and are able to exit this state when they are brought back into an adherent 2D culture [30]. The primary murine cells dissociated from the myospheres were pre-myogenic and multipotent, as shown by their myogenic, adipogenic, and osteogenic potential [29], although incomplete cell fusion of mouse myosphere-derived cells was described as well [33]. Here, after dissociation from the spheroids, the C2C12 cells and PMCs attached easily and rapidly as soon as an appropriate surface was provided and were able to re-enter the cell cycle and start to proliferate again.

We isolated cells from muscles of very young piglets, when the myogenic progenitor cells are still in a very dynamic growth phase and mostly did not settle into their stem cell niche, but are still cycling [73]. Due to this high plasticity of porcine myogenic (stem) cells, they retain a more immature phenotype in myospheres compared to C2C12, which rapidly differentiate when reaching confluency [74]. The finding that substantial F-Actin and MHC expression was only found in C2C12-derived spheroids supports this. This corresponds to previous data with a different cultivation system, where Myosin expression was reported to start at Day 3 and to peak at Days 9–10 in C2C12 spheroids [30]. For PMC-derived spheroids, longer cultivation might be necessary to detect the late differentiation markers, such as MHC. This was also hypothesized by Westerman and colleagues [29], when working with primary murine cells, which exhibited a more primitive spheroid phenotype. Multinucleated syncytia are characteristic for skeletal muscle maturation and it was hypothesized that superior aggregation of cells will also favor syncytia formation [31]. Thus, their frequent presence in C2C12-derived spheroids points to myogenic differentiation of cells already during spheroid cultivation and might be associated with a higher packing density. In PMC-derived spheroids we did not observe syncytia formation.

The differentiation potential of C2C12 as well as PMC was conserved during spheroid culture in our study. When cells dissociated from spheroids were allowed to differentiate in 2-dimensional culture under serum-reduced conditions, cells fused into multinucleated myotubes. Additionally, the early differentiation marker MyoG was found in cell nuclei within myotubes as well as in mononuclear cells. This was even true for PMC, although these cells were already passaged three times and seeded for differentiation assay up to 2 weeks after initial isolation. It is well known that cells of the C2C12 line differentiate easily and in a superior way into the myogenic lineage compared to primary cells (especially after passaging). Therefore, it is not surprising that a greater number of elongated cells (Des+, Myosin HC+) and MyoG+ cells seem to be present in C2C12 cultures. It was hypothesized that the presence of serum might play a role in the differentiation potential of cells dissociated from murine spheroids [29], since [33] described problems with cell fusion after cultivating myospheres in medium containing FBS. In contrast, [29] observed cell fusion into multinucleated myotubes when using serum-free conditions during spheroid cultivation. Nevertheless, we used serum during 3D as well as during 2D culturing and were able to achieve cell fusion of murine and porcine myogenic cells.

## 5. Conclusions

We present the scaffold-free formation and cultivation of myospheres in a mid-scale bioreactor and compare a conventional myogenic cell line (C2C12) to primary porcine muscle cells (PMCs). The production of porcine myospheres has not been described before. Spheroids from PMCs and C2C12 cells can be cultured for at least 1 week. For long-time cultivation, further optimization of the conditions might be necessary. Myogenic cells exhibited no apoptosis or cell death, retained their myogenic identity during spheroid formation, and were able to form myotubes afterwards. From our experience in working with the hanging drop method, we think that the bioreactor system generates more homogenous myospheres, which can be handled more easily and cultivated longer. In addition, the technology allows upscaling the process in a better and more reproducible manner.

We are convinced that the system described here is suitable for other sensitive primary cells as well and enhances the possibilities to explore the stemness and differentiation potential of myogenic cells in 3-dimensional cell cultures.

## Figures and Tables

**Table 1:** Exemplary overview on studies involving myogenic spheroids generated with different technologies.

Myosphere Culture Method	Biomaterial	Species	Cell Type	Cultivation Duration	Reference(s)
Static suspension culture (culture flask/plate)	None	Mouse	C2C12 myoblast cell line	>20 days	[30]
				8 days	[31]
			Primary MDC	Several months	[27,29,33]
		Human	Embryonic stem cell line D3	7 days	[40]
			Primary MDC	Up to 5 months	[34]
				1–2 weeks	[35,36]
		Dog	Primary MDC	14 days	[37]
	Hydrogel (hyaluronic acid/fibrinogen)	Human	Vascular smooth muscle cell line (SMCs)	14 days	[32]
Hanging drop	None	Mouse	C2C12 myoblast cell line	1 week	[41]
		Mouse	Primary MDC	1 week	[41]
		Human	Embryonic stem cell line D3	7 days	[40]
Stirrer flask	Microcarrier (polystyrene or dextran)	Bovine	Primary MDC	8 days	[38]
Rotary cell culture system	None	Mouse	C2C12 myoblast cell line	8 days	[31]
Bioreactor	Cylindric sponge (hyaluron)	Rat	Primary MDC	Up to 25 days	[39]

**Table 2:** Parameters for suspension culture using the CERO 3D Incubator and Bioreactor.

	C2C12		PMCs	
	Inoculation Phase	Culture Phase	Inoculation Phase	Culture Phase
Rotation period	2 s	1 s	1 s	1 s
Rotation speed	80 rpm	80 rpm	80 rpm	80 rpm
Rotation pause	0 s	0 s	0 s	2 s
Protocol duration	12 h	7 d	6 h	7 d

## Data Availability

The datasets used and/or analysed during the current study are available from the corresponding author on reasonable request.
